# Advances in Understanding and Managing Floating Knee Injuries: A Comprehensive Review

**DOI:** 10.7759/cureus.57122

**Published:** 2024-03-28

**Authors:** Sankalp Yadav, Gautam Rawal

**Affiliations:** 1 Medicine, Shri Madan Lal Khurana Chest Clinic, New Delhi, IND; 2 Respiratory Medical Critical Care, Max Super Speciality Hospital, New Delhi, IND

**Keywords:** tibia, femur, rehabilitation, dislocation, fracture, floating knee injury

## Abstract

Fractures of the ipsilateral tibia and femur, frequently combined with soft tissue damage and dislocations, describe floating knee injuries, a complicated orthopedic condition. Epidemiological data suggest that floating knee injuries account for a small but significant proportion of traumatic orthopedic injuries, with a higher incidence observed in younger males engaged in high-risk activities. Anatomically, floating knee injuries involve fractures of the femur and tibia, ligamentous disruptions, and soft tissue damage, contributing to the complexity and severity of these injuries. An extensive analysis of floating knee injuries is given in this paper, including information about epidemiology, anatomy, pathophysiology, categorization, management approaches, complications, prognosis, and current and upcoming developments.

## Introduction and background

Concomitant femur and ipsilateral tibia fractures, frequently combined with dislocations and significant soft tissue trauma, characterize floating knee injuries, a complicated and devastating category of traumatic orthopedic injuries [1]. Blake and McBryde originally recorded the term "floating knee" in 1975 [2]. High-velocity events like automobile crashes, falls from great heights, and sports-related accidents that include strong direct or indirect forces applied to the lower extremities are usually the cause of these injuries [3]. The expression "floating knee" describes the dislocation of the lower limb's proximal and distal portions from the knee joint, which poses a significant management challenge for orthopedic surgeons because of the particular anatomical and biomechanical factors involved [2].

Within the scope of orthopedic medicine, floating knee injuries hold profound clinical significance owing to their complexity, severity, and potential for catastrophic outcomes [4]. The simultaneous involvement of two major weight-bearing bones of the lower extremity, coupled with the disruption of the knee joint, poses significant challenges in terms of diagnosis, treatment planning, and postoperative rehabilitation [5]. Moreover, the considerable forces involved in the mechanism of injury often lead to associated injuries, including neurovascular compromise, compartment syndrome, and visceral injuries, further complicating the clinical picture and necessitating a multidisciplinary approach to management [6].

The impact of floating knee injuries on patient outcomes cannot be overstated. Beyond the immediate physical trauma and pain, individuals afflicted with these injuries often experience profound functional impairment, long-term disability, and a diminished quality of life [7]. The psychological toll of enduring such a traumatic event, coupled with the uncertainty surrounding recovery and rehabilitation, further exacerbates the burden on patients and their families [8]. Consequently, there exists an urgent need for clinicians and researchers to deepen their understanding of floating knee injuries, explore innovative treatment modalities, and refine existing therapeutic strategies to mitigate morbidity and improve long-term prognosis.

The aim of this review is to highlight this infrequently reported clinical condition and provide information on its management and complications, along with some details about recent developments.

## Review

Epidemiology and etiology

Prevalence

Floating knee injuries are relatively uncommon but represent a significant subset of traumatic orthopedic injuries [9]. While precise epidemiological data may vary across different populations and regions, studies suggest that floating knee injuries account for approximately 0.3% to 2% of all fractures [10]. The incidence tends to be higher in younger age groups, particularly adolescents and young adults, likely due to increased participation in high-risk activities, such as sports and motor vehicle-related incidents [3].

Demographically, males are disproportionately affected by floating knee injuries, with a male-to-female ratio ranging from 2:1 to 5:1. This gender disparity is attributed to higher rates of participation in physically demanding activities and occupations among males, predisposing them to a greater risk of traumatic injuries [3].

Furthermore, certain occupational groups, such as construction workers, military personnel, and athletes involved in contact sports, are at increased risk of sustaining floating knee injuries due to the nature of their activities and exposure to high-impact forces [11-13]. Additionally, socioeconomic factors and environmental conditions may influence the incidence of these injuries, with disparities observed in urban versus rural settings and among different socioeconomic strata.

Etiology

Floating knee injuries typically result from high-energy trauma involving significant axial loading, torsional forces, or direct impact on the lower extremities. The most common causes and mechanisms behind these injuries are listed below.

Motor vehicle accidents: Floating knee injuries are a common consequence of collisions involving cars, motorcycles, or other motorized vehicles because of the high-velocity forces that are transferred to the lower limbs during impact [14]. People are more vulnerable to femur and tibia fractures due to a combination of abrupt stops, severe trauma, and crushing forces. These fractures are frequently accompanied by ligamentous damage and dislocations.

Falls from height: Falls from significant heights, such as scaffolding, ladders, or elevated surfaces, can lead to floating knee injuries due to the abrupt impact upon landing. The compressive forces generated upon impact, coupled with the energy dissipation through the lower extremities, can result in fractures of the femoral and tibial shafts, as well as intra-articular injuries to the knee joint [14,15].

Sports-related incidents: Participation in high-impact sports, including football, soccer, skiing, and extreme sports, predisposes athletes to floating knee injuries. Collisions with opponents, awkward landings, and high-velocity falls during sporting activities can impart significant forces to the lower limbs, resulting in fractures, dislocations, and ligamentous injuries [13].

Occupational injuries: Individuals engaged in physically demanding occupations, such as construction workers, laborers, and military personnel, are at heightened risk of sustaining floating knee injuries due to exposure to hazardous working conditions and heavy machinery. Accidents involving falls, crush injuries, or machinery malfunctions can lead to traumatic fractures and dislocations of the lower extremities.

Violent trauma: Assaults, physical altercations, and acts of violence may also precipitate floating knee injuries, particularly in urban areas with higher rates of interpersonal violence. The intentional application of blunt force trauma or the use of weapons can result in severe musculoskeletal injuries, including fractures and dislocations of the knee joint.

Anatomy and pathophysiology

Floating knee injuries entail complex damage to various anatomical structures within the lower extremity, which are listed below.

Bones

Femur: The femur, or thigh bone, is the longest and strongest bone in the human body [16]. Fractures of the femoral shaft or condyles are common in floating knee injuries, often occurring due to axial loading or direct trauma.

Tibia: The tibia, or shinbone, is the larger of the two lower leg bones and bears most of the body's weight [17]. Fractures of the tibial shaft or plateau are frequently seen in conjunction with femoral fractures in floating knee injuries [18].

Knee Joint

Articular surfaces: The patella, tibial plateau, and femoral condyles articulate to allow for seamless lower limb movement, making up the knee joint. Intra-articular fractures or dislocations can happen with floating knee injuries, which can cause cartilage injuries and joint instability.

Ligaments: The anterior cruciate ligament (ACL), posterior cruciate ligament (PCL), medial collateral ligament (MCL), and lateral collateral ligament (LCL) are among the ligaments that support the knee joint [19]. These ligaments are susceptible to injury during high-energy trauma, resulting in ligamentous laxity or tears [20].

Soft Tissues

Muscles and tendons: The muscles and tendons surrounding the knee joint play a crucial role in movement and stability [21]. In floating knee injuries, these soft tissues may sustain contusions, strains, or avulsion injuries, contributing to pain and functional impairment.

Menisci: The medial and lateral menisci are fibrocartilaginous structures that serve to cushion and stabilize the knee joint [21]. Traumatic injury to the menisci can result in tears, leading to pain, swelling, and mechanical symptoms such as locking or clicking.

Pathophysiological changes

Floating knee injuries involve a cascade of pathophysiological changes that disrupt the integrity and function of the lower extremity. Key alterations are mentioned below.

Fractures

Femoral and tibial fractures: High-energy trauma can result in femur and tibia comminuted, displaced fractures, frequently accompanied by soft tissue damage and periarticular involvement [6].

Intra-articular fractures: Fractures in the knee joint's articular surfaces can cause instability, cartilage degradation, and a higher chance of developing post-traumatic arthritis by upsetting the joint's congruity [22].

Dislocations

Dislocations of the knee joint, in which the femur and tibia move out of alignment, are a possible consequence of floating knee injuries. Dislocations have the potential to jeopardize neurovascular structures; hence, immediate reduction and stabilization are required to stop additional damage.

Damage to Soft Tissue

Damage to the ligaments: ACL, PCL, MCL, and LCL tears or avulsions can result from the high-energy stresses associated with floating knee injuries, impairing knee stability and raising the possibility of recurrence.

Muscle and tendon injuries: Contusions, strains, or tears of the muscles and tendons surrounding the knee joint are common in floating knee injuries, contributing to pain, swelling, and functional impairment.

Vascular and neurological compromise: Severe trauma may result in vascular or neurological compromise, manifesting as arterial injury, compartment syndrome, or peripheral nerve damage, necessitating prompt evaluation and intervention [23].

Classification and severity grading

A number of classification schemes have been put out to group floating knee injuries according to the severity and anatomical features of the injury. There are two widely used classification schemes.

Blake and McBryde began by classifying injuries according to the parts of the body in which they occurred. Three categories were established: type I, type II-A, and type II-B. The two long bones' two shafts are impacted by type I fractures. Type II-B fractures can affect the hip or ankle joints, whereas type II-A fractures impact the knee joint [4].

There are two classification systems that are specifically made for pediatric fractures: Letts-Vincent and Bohn-Durbin. They start by classifying the fracture location according to its closure or openness. Fractures are categorized into types A-E by Letts-Vincent. Two closed diaphyseal fractures make up a type A fracture. Two closed fractures, one diaphyseal and the other metaphyseal, are present in type B injuries. Two closed fractures, one diaphyseal and the other epiphyseal, are included in type C injuries. Whereas type E fractures entail both fractures being open; type D injuries feature at least one open fracture [3].

Three types make up the Bohn-Durbin classification system. Type II injuries are juxta-articular, which means they happen close to a joint; type III fractures have an epiphyseal component, which indicates involvement close to the growth plate; and type I fractures involve double shaft fractures [24].

Three forms of floating knee injuries are distinguished by the Fraser classification, which was first presented by Fraser et al. in 1979 [25]. Type I: femur and tibia fractures that are ipsilateral and do not involve the intra-articular space. Type II: femur and tibia fractures that are ipsilateral and affect the intra-articular space. Type III: contralateral femur and tibia fractures, frequently linked to trauma to the pelvis or spine. Type IV: fracture of one bone and dislocation of the ipsilateral knee or ankle joint.

Ran et al. have proposed a change to this categorization that considers the complexity of the articular fracture and how it affects the patella [26]. These classifications are intended to aid in prognosis-setting, as functional recovery is better and fewer complications result from a priori diaphyseal fractures than from those affecting the joint. Nevertheless, neither of these classes takes into consideration accompanying soft-tissue injuries or ligament injuries, nor does Ran's classification account for diaphyseal fractures connected to a fractured patella.

Severity Grading Systems

Severity grading systems help clinicians assess the extent of injury and guide treatment decisions by stratifying patients into different risk categories and predicting outcomes. These systems may incorporate various parameters, including fracture morphology, soft tissue injury, neurovascular compromise, and associated injuries. Severity grading systems facilitate communication among healthcare providers, aid in treatment planning, and inform prognostication. Examples of severity grading systems used in the management of floating knee injuries include the Gustilo-Anderson classification for open fractures, which categorizes open fractures based on the size of the wound, extent of soft tissue damage, and degree of contamination, and Schatzker classification for tibial plateau fractures, which stratifies tibial plateau fractures based on the pattern of articular involvement and severity (Table 1) [27,28].

**Table 1:** Severity grading systems used in the management of floating knee injuries.

Gustilo-Anderson classification for open fractures	Schatzker classification of tibial plateau fractures
Type I: Clean wound less than 1 cm in size.	Type I: Split fracture of the lateral plateau.
Type II: Wound 1-10 cm in size with moderate soft tissue damage.	Type II: Split fracture of the medial plateau.
Type III: Wound greater than 10 cm in size with extensive soft tissue damage and contamination, further subdivided into IIIA, IIIB, and IIIC based on severity.	Type III: Depression fracture of the lateral plateau.
	Type IV: Depression fracture of the medial plateau.
	Type V: Bicondylar fracture with both medial and lateral plateau involvement.
	Type VI: Complex fracture pattern involving the entire tibial plateau.

Injury Severity Score

A scoring method known as the Injury Severity Score (ISS) uses the Abbreviated Injury Scale (AIS) ratings allocated to each anatomical region to determine the total severity of various traumatic injuries. Higher scores on the ISS, which goes from 0 to 75, indicate more serious injuries and a higher probability of death [29].

By employing classification and severity grading systems, clinicians can stratify floating knee injuries according to their anatomical characteristics, associated injuries, and severity, facilitating appropriate treatment selection, prognostication, and multidisciplinary care coordination. These systems serve as valuable tools in the management of complex orthopedic injuries and contribute to improved outcomes for patients with floating knee injuries.

Clinical presentation

Floating knee injuries present with a range of signs and symptoms that reflect the severity of the trauma and the extent of musculoskeletal involvement. Common clinical manifestations include the following.

Pain

Patients typically experience significant pain in the affected lower limb, localized to the site of fractures and soft tissue injuries. The pain may be exacerbated by weight-bearing activities or movement of the knee joint [3].

Swelling

Edema and swelling are commonly observed following floating knee injuries, resulting from inflammation and soft tissue trauma. There could be swelling only in the knee joint or all the way down the lower limb.

Deformity

Abnormal alignment of the femur and tibia, limb shortening, or angular abnormalities may be indicative of a gross lower limb deformity. Physical examination can reveal obvious deformities caused by fractures that are displaced or dislocated.

Instability

Floating knee injuries often lead to instability of the knee joint, characterized by a sense of giving way, laxity, or abnormal mobility. Ligamentous injuries or joint dislocations contribute to knee instability and may impair weight-bearing and functional activities [30].

Impaired Mobility

Reduced range of motion and impaired mobility are common sequelae of floating knee injuries, attributable to pain, swelling, joint stiffness, and mechanical restrictions. Patients may experience difficulty walking, standing, or performing activities of daily living.

The importance of clinical examination and diagnostic imaging

A thorough clinical examination is essential for evaluating patients with suspected floating knee injuries and guiding subsequent diagnostic and treatment decisions. Key components of the clinical assessment include the following.

History Taking

Comprehensive history-taking should include details regarding the mechanism of injury, onset and duration of symptoms, associated symptoms (e.g., neurovascular deficits), past medical history, and any predisposing factors (e.g., previous trauma and underlying musculoskeletal conditions).

Physical Examination

A systematic physical examination should be conducted to assess for signs of trauma, deformity, swelling, tenderness, instability, range of motion, neurovascular status, and functional impairment. The stability and alignment of the knee joint, the feel of bony landmarks, and the evaluation of ligamentous integrity all require special attention.

Imaging Diagnostics

To confirm the diagnosis, describe the severity of the injury, and direct treatment planning, diagnostic imaging is essential. The following imaging techniques are frequently employed in the assessment of floating knee injuries [3].

Radiograph/X-ray: Plain radiographs of the knee, femur, and tibia provide an initial assessment of bony alignment, fracture patterns, and associated injuries.

Computed tomography (CT): To help in surgical planning and fracture classification, CT imaging provides precise visibility of complex fracture patterns, intra-articular involvement, and bony landmarks [31].

Magnetic resonance imaging (MRI): MRI is valuable for assessing soft tissue injuries, ligamentous integrity, meniscal tears, and intra-articular pathology, particularly in cases of suspected ligamentous or cartilaginous injury [32].

Management strategies

Conservative Management

Conservative management may be considered for stable floating knee injuries with minimal displacement and soft tissue involvement [33,34]. Treatment typically involves immobilization with a brace or cast, non-weight-bearing or partial weight-bearing, and early mobilization as tolerated. Physical therapy and rehabilitation exercises are initiated to improve the range of motion, strengthen muscles, and facilitate functional recovery [35]. Close monitoring of clinical progress and serial imaging studies may be warranted to assess fracture healing and ensure the stability of the knee joint.

Surgical Intervention

Surgical intervention is often indicated for unstable floating knee injuries, intra-articular fractures, displaced fractures, ligamentous instability, and cases refractory to conservative management [3].

Surgical options may include open reduction and internal fixation (ORIF), intramedullary nailing, external fixation, arthroscopic-assisted reduction and fixation, ligament reconstruction, and joint reconstruction procedures [36].

The choice of surgical approach and technique depends on factors such as fracture pattern, soft tissue condition, associated injuries, the patient's age, and the surgeon's expertise.

Surgical goals include anatomical reduction of fractures, restoration of joint alignment and stability, preservation of soft tissue vascularity, and early mobilization to prevent complications such as stiffness and joint contractures.

Rehabilitation Protocols

Rehabilitation protocols play a crucial role in optimizing functional outcomes and facilitating a return to pre-injury activities [37]. Early mobilization and range-of-motion exercises are initiated postoperatively to prevent stiffness and promote joint mobility. Progressive weight-bearing and strengthening exercises are introduced as tolerated, focusing on restoring muscle strength, balance, and proprioception. Functional activities and sports-specific training are incorporated into the rehabilitation program to improve agility, coordination, and functional capacity. Close collaboration between orthopedic surgeons, physical therapists, and other members of the healthcare team is essential to ensuring coordinated care and achieving optimal rehabilitation outcomes.

Factors Influencing Treatment Decisions

Type and severity of injury: The nature and severity of the fracture pattern, the presence of intra-articular involvement, ligamentous instability, and associated injuries dictate the choice of treatment modality [38].

Patient's age: Younger patients may tolerate more aggressive surgical interventions and have better healing potential compared to older individuals with comorbidities.

Overall health: Patients with pre-existing medical conditions, such as osteoporosis, diabetes, or cardiovascular disease, may have increased surgical risks and require tailored treatment approaches.

Functional goals: Treatment decisions should align with the patient's functional goals, lifestyle, and expectations for recovery, emphasizing the importance of shared decision-making and patient-centered care.

Surgeon's expertise: The surgeon's experience, technical skills, and familiarity with various treatment modalities influence the selection of surgical techniques and approaches.

Complications

A number of possible consequences linked to floating knee injuries have the potential to seriously affect patient outcomes and quality of life [3].

Compartment Syndrome

Increased pressure inside the lower extremity muscle compartments is the hallmark of compartment syndrome, which can result from high-energy trauma and significant soft tissue injury in floating knee injuries. If left untreated, compartment syndrome can lead to problems that require an emergency fasciotomy, tissue ischemia, and nerve damage that could endanger limbs [39].

Neurovascular Injury

Floating knee injuries may cause damage to neurovascular structures, including peripheral nerves, arteries, and veins. Neurovascular compromise can lead to sensory deficits, motor dysfunction, vascular insufficiency, and limb ischemia if not promptly recognized and managed [40].

Malunion and Nonunion

Improper fracture reduction, delayed union, or inadequate fixation can result in malunion or nonunion of fractures in floating knee injuries. While nonunion makes it more difficult to cure fractures and may require revision surgery or bone grafting, malunion can result in limb deformity, altered biomechanics, and decreased function [3].

Post-traumatic Arthritis

Patients with floating knee injuries are at risk of developing post-traumatic arthritis because of intra-articular involvement and articular surface injury [41]. Joint stiffness, degenerative cartilage, and persistent discomfort can severely limit joint function and range of motion, calling for conservative measures or, in more extreme situations, joint replacement surgery.

Infection

The risk of infection in floating knee injuries is increased by open fractures, severe soft tissue trauma, and surgical procedures [41].

Deep tissue infections, osteomyelitis, and septic arthritis can lead to prolonged hospitalization, delayed rehabilitation, and compromised outcomes if not promptly diagnosed and treated with antibiotics and surgical debridement.

Prognosis and functional outcomes

There has been limited research conducted on the long-term outcomes and functional results of floating knee injuries [42]. Injuries to the floating knee have different long-term prognoses and functional results depending on the severity of the injury, type of therapy administered, age of the patient, general health, and compliance with rehabilitation.

Conservative Management

Patients managed conservatively with immobilization, protected weight-bearing, and physical therapy may achieve satisfactory outcomes in cases of stable fractures with minimal displacement. However, prolonged immobilization and delayed weight-bearing may lead to muscle weakness, joint stiffness, and functional limitations [42].

Surgical Intervention

Surgical management of floating knee injuries aims to achieve anatomical reduction, stable fixation, and early mobilization to optimize outcomes [36]. Patients undergoing timely surgical intervention with appropriate fracture fixation, soft tissue reconstruction, and comprehensive rehabilitation protocols generally have better functional outcomes and lower rates of complications compared to non-operative management.

Rehabilitation

The success of rehabilitation programs for floating knee injuries is critical for restoring mobility, strength, and functional independence. Early mobilization, range of motion exercises, progressive weight-bearing, and functional training are essential components of rehabilitation protocols aimed at improving joint function, proprioception, and return to pre-injury activities [37].

Joint Replacement

In cases of severe post-traumatic arthritis or irreparable joint damage, total knee arthroplasty (TKA) may be considered to alleviate pain, restore joint function, and improve quality of life. TKA can provide durable pain relief and functional improvement, although the decision to undergo joint replacement surgery should be carefully weighed against the patient's age, activity level, and overall health status [43].

Recent advances and future directions

Minimally Invasive Surgical Techniques

Recent advancements in minimally invasive surgical techniques, such as arthroscopy-assisted reduction and fixation, have revolutionized the management of floating knee injuries. Arthroscopic guidance allows for the precise reduction of intra-articular fractures, minimizes soft tissue trauma, and facilitates early mobilization and rehabilitation [44].

Minimally invasive approaches, including percutaneous screw fixation, intramedullary nailing, and percutaneous plate osteosynthesis, offer potential benefits such as reduced surgical morbidity, shorter hospital stays, and faster recovery times compared to traditional open procedures.

Biomechanical Innovations

Biomechanical studies and advancements in implant design have led to the development of novel fixation devices and surgical techniques tailored to the specific biomechanical demands of floating knee injuries [45].

Biodegradable implants, locking plates, intramedullary nails with angular stability, and hybrid fixation constructs have been introduced to provide greater stability, enhanced load-sharing capabilities, and improved fracture healing while minimizing implant-related complications [46].

Novel Rehabilitation Strategies

Emerging rehabilitation strategies focus on early mobilization, functional training, and patient-centered approaches to optimize recovery and functional outcomes in individuals with floating knee injuries [47].

Advanced rehabilitation technologies, such as virtual reality-based training, robotic-assisted therapy, and sensor-assisted gait analysis, offer innovative approaches to enhance neuromuscular re-education, proprioception, and motor control in the rehabilitation process [48].

Biological Augmentation and Tissue Engineering

Ongoing research efforts in biological augmentation and tissue engineering aim to harness the regenerative potential of stem cells, growth factors, and biomaterials to enhance fracture healing, soft tissue repair, and joint regeneration in floating knee injuries [49].

Biological adjuncts, such as platelet-rich plasma (PRP), mesenchymal stem cell therapy, and tissue-engineered scaffolds, hold promise for promoting tissue regeneration, reducing inflammation, and accelerating recovery in complex musculoskeletal injuries [50].

Patient-Specific Treatment Approaches

Advances in imaging technology, computational modeling, and personalized medicine enable clinicians to tailor treatment strategies to individual patient characteristics, injury patterns, and functional goals [51].

Patient-specific implants, custom surgical guides, and computer-assisted navigation systems facilitate precise surgical planning, implant positioning, and intraoperative decision-making, leading to improved surgical outcomes and patient satisfaction [52].

Multidisciplinary Care and Collaborative Research

Multidisciplinary care models integrating orthopedic surgeons, trauma specialists, physical therapists, biomechanical engineers, and rehabilitation specialists facilitate comprehensive management and holistic approaches to floating knee injuries.

Collaborative research endeavors focusing on epidemiology, biomechanics, outcomes assessment, use of newer technologies (e.g., digitization, information and communication technology (ICT), internet of things (IoT), cloud technology, artificial intelligence (AI), cloud computing, known additive manufacturing (AM), and big data and cyber systems), and translational research are vital for advancing our understanding of floating knee injuries, refining treatment algorithms, and optimizing clinical outcomes [53].

Recommendations

To address the challenges posed by floating knee injuries effectively, the following recommendations are proposed.

Enhanced Education and Awareness

Clinicians should be educated about the recognition, diagnosis, and management of floating knee injuries, emphasizing the importance of early intervention and multidisciplinary collaboration.

Advancements in Surgical Techniques

Continued research and development of minimally invasive surgical techniques, biomechanical innovations, and implant technologies can improve surgical outcomes, reduce complications, and expedite rehabilitation.

Innovations in Rehabilitation

Novel rehabilitation strategies focusing on early mobilization, functional training, and patient-centered care can optimize functional outcomes and facilitate a return to pre-injury activities.

Multidisciplinary Approach

Multidisciplinary teams comprising orthopedic surgeons, trauma specialists, physical therapists, and rehabilitation professionals should collaborate to provide comprehensive care and support throughout the continuum of care.

Research Initiatives

Ongoing research efforts should be directed toward understanding the pathophysiology of floating knee injuries, evaluating treatment outcomes, identifying prognostic factors, and exploring innovative therapeutic modalities to improve patient outcomes and reduce morbidity.

## Conclusions

The review discussed floating knee injuries, which involve fractures of the femur and ipsilateral tibia, often accompanied by dislocations and soft tissue trauma. These injuries are complex and relatively uncommon but can lead to significant morbidity, especially in active individuals. The anatomical structures involved include bones, ligaments, tendons, and soft tissues, making diagnosis and classification crucial for effective treatment. Treatment options range from conservative approaches like immobilization and physical therapy to surgical interventions such as fracture fixation and joint replacement. Complications like compartment syndrome, neurovascular injury, and post-traumatic arthritis may arise, affecting long-term prognosis and functional outcomes, which depend on various factors, including injury severity, treatment, patient age, health, and rehabilitation compliance.
